# Letter to the Editor on: “Safety and Efficacy of Poly-L-Lactic Acid Filler (Gana V vs. Sculptra) Injection for Correction of the Nasolabial Fold: A Double-Blind, Non-Inferiority, Randomized, Split-Face Controlled Trial”

**DOI:** 10.1007/s00266-023-03736-x

**Published:** 2023-11-22

**Authors:** Sabrina Fabi, Daniel Bråsäter

**Affiliations:** 1 Cosmetic Laser Dermatology, 9339 Genesee Ave #300,, San Diego, California USA; 2 Galderma, Uppsala, Sweden

*Level of Evidence V* This journal requires that authors assign a level of evidence to each article. For a full description of these Evidence-Based Medicine ratings, please refer to the Table of Contents or the online Instructions to Authors www.springer.com/00266.

With great interest we have read the article published by Han et al. titled “Safety and Efficacy of Poly-L-Lactic Acid Filler (Gana V vs. Sculptra) Injection for Correction of the Nasolabial Fold: A Double-Blind, Non-Inferiority, Randomized, Split-Face Controlled Trial” [1]. A study officially titled “*Clinical Study for the Safety and Effectiveness of Use of an Injectable Medical Device GANA V® for Facial Aesthetic”* (Clinicaltrials.gov ID: NCT05215054), with the described purpose to assess the effectiveness and safety of Gana V, a poly-L-lactic acid filler for the aesthetic treatment of nasolabial folds, in comparison with Sculptra (poly-L-lactic acid (PLLA), Galderma).

There are several misleading incorrect statements not supported by science nor literature and shortcomings existing in the Han et al. Non-Inferiority, Randomized, Split-Face Controlled Trial that should be noted.

Han et al. describe Gana poly-lactic acid (Gana V, GCS Co.) as a US FDA-approved PLLA filler which is a false statement. Of the two PLLA fillers included in the trial, only Sculptra poly-L-lactic acid (PLLA; Sculptra, Galderma) filler is US FDA approved [2]. Han et al. also state that “Sculptra requires preparation hours before injection, “Gana V could be mixed and used immediately at the time of injection” which is an incorrect statement [3, 4], lacking scientific support [5] and contributes to misleading and incorrect communication. Gana poly-lactic acid (Gana V, GCS Co.) per manufacturer’s instruction has at least 1 hour and 10 minutes of preparation time (shaking time 10 minutes + 1 hour of standing time) per instructions for use [5] with a lack of evidence for immediate use. The fact is the opposite than stated in the published work, Sculptra poly-l-lactic acid can be used immediately after reconstitution published by Baumann et al. (2020) [3, 4]. Sculptra’s 8mL immediate reconstitution has also been evaluated in several studies (Palm et al. 2021) [7, 8]) and in a pivotal clinical trial (NCT04124692) gaining US FDA approval [2].

To demonstrate non-inferiority (NI), common practice is that after study completion, a confidence interval (CI) for the difference between the two agents (devices) is constructed. This interval should lie entirely on the positive side of the pre-specified NI margin to conclude non-inferiority [6]. Han et al. are not reporting the CI of the difference (in change from baseline on the WSRS in this case) between the devices evaluated in the published work. Instead, Han et al. conclude “that Gana V was non-inferior to Sculptra with respect to the correction of NLFs” based on a test and a non-significant p-value which is normally not an acceptable method of concluding NI. However, it is also unclear what NI margin was used for conclusion of non-inferiority, since the margin of 15% mentioned in the Methods section seems to refer to a proportion and not a mean change from baseline. Furthermore, Han et al. also report and describe very small changes in WSRS for Gana V which are claimed to be improvements. Although statistically significant, changes this small can hardly be considered as clinically relevant and may suggest that the study is over-powered for this type of comparison. The study outcomes reflected in the article are not balanced to Han et al.’s conclusions.

The study title indicates that this study is a non-inferiority, randomized, split-face controlled trial, head-to-head, comparison of poly-L-lactic acid (Sculptra 150mg PLLA, Galderma) and poly-L-lactic acid (Gana V, 210mg PLLA, GCS Co.) for the correction of Nasolabial Folds (NLF), suggesting that two PLLA filler treatments used per manufacturer instruction are compared with the objective to show non-inferiority. The reported results and study conclusions presented by Han et al. are based on several study design flaws, describing a study not conducted per clinical practice, nor per manufacturer’s instruction for use, and reported study outcomes not reported per guidelines to describe non-inferiority [6]. Han et al. empower the strength and high reliability of the non-inferiority study design, but the majority of the trial design’s flaws and limitations are not objectively discussed to reflect that the study does not comply with respective PLLA filler manufacturer’s instructions for use, recommendations or adhere to published clinical trial data [4, 5, 9]. The non-inferiority study design presented explores a low PLLA injection volume (1 mL) with a limited number of sessions (1 + 1) not spaced according to previous trial data [4, 5, 9, 10] and is therefore not reflecting clinical practice, significant caveats and limitations to consider but not objectively addressed in the discussion nor the conclusion.

Safety and Efficacy are corner stones to evaluate a risk–benefit profile of any investigational drug or medical device and of great importance for practitioners and patients. Han et al. report that Gana V and Sculptra have “similar safety profile patterns” and “in terms of local adverse reactions, we found no difference between Gana V and Sculptra.” The safety profile of Gana V treatment arm is consistently worse than the safety profile reported for the Sculptra treatment arm with the exception of bruising. Han et al. report approximately ≈19% (18.7%) more injection-related adverse events for Gana V than Sculptra in the article’s full analysis set, beside adverse reactions captured in the supplementary material, but treatment arm not disclosed (e.g., one nodule and two skin thickening/induration). The safety profile of the full analysis set also reveals a > twofold higher incidence rate of lumps/bumps and a > fourfold higher incidence rate of induration/firmness-related Gana V injections. Also, note that Han et al. [1] in table 4 list two [2] injection sites bruising related to Sculptra injections converted to 52.7% which is incorrect and the correct percentage is 3.6%.

Overall, Han et al. present a trial design more suggesting an experimental and exploratory study design rather than a non-inferiority trial, describing and reporting on differences between two 1mL poly-L-lactic acids in humans.

Besides the limitations and errors, Han et al. are not reporting on the CI difference for changes from baseline on the Wrinkle Severity Rating Scale (WSRS) to describe non-inferiority between Gana V and Sculptra. Furthermore, the non-inferiority calculations are based on a test and p-value that is not significant and report on very small changes in WSRS considered as not clinically relevant.

A published work which includes several incorrect and unsupported statements and reporting on a trial design with multiple flaws, limitations and caveats with a non-inferiority calculation not per guidelines, not significant and findings not clinically relevant disqualify the study as a classical non-inferiority trial and from concluding non-inferiority between Gana V and Sculptra PLLA.
